# The relationship between exercise commitment and psychological detachment from academic activities among Chinese college students: the chain mediating roles of self-identity and sense of belonging

**DOI:** 10.3389/fpsyg.2026.1810232

**Published:** 2026-07-30

**Authors:** Weiguo Li, Jun Xiang, YeZhou Guo

**Affiliations:** 1 School of Physical Education and Health, Zhaoqing University, Zhaoqing, China; 2 School of Athletic Performance, Shanghai University of Sport, Shanghai, China

**Keywords:** exercise commitment, mediating role, psychological detachment, self-identity, sense of belonging

## Abstract

**Background:**

Mental health concerns among college students have received increasing attention in recent years. Psychological detachment—defined as mentally disengaging from academic-related thoughts during non-study time—is typically conceptualized as a recovery-related experience; however, its role in academic contexts may be context-dependent.

**Objective:**

This study examines the association between exercise commitment and psychological detachment and explores the roles of self-identity and sense of belonging within a multi-level framework.

**Methods:**

A stratified multi-site sampling strategy combined with classroom-based recruitment was used to survey 850 Chinese college students (aged 17–25 years; 48.82% male). Measures included the Exercise Commitment Scale, the psychological detachment subscale of the Recovery Experience Questionnaire (REQ), the Self-Identity Scale, and the Sense of Belonging Inventory. Data were analyzed using Pearson correlations and Hayes’ PROCESS macro (Model 6) with bias-corrected bootstrap resampling (5,000 samples).

**Results:**

Exercise commitment was negatively associated with psychological detachment (*r* = −0.501, *p* < 0.001). Indirect associations via self-identity (37.02%), sense of belonging (14.19%), and their sequential association (13.84%) were statistically significant. Alternative model specifications produced comparable results, suggesting that the proposed mediation structure is not uniquely supported.

**Conclusion:**

Higher exercise commitment was associated with lower psychological detachment in academic contexts. The functional meaning of this association remains context-dependent. These findings should be interpreted as theoretically informative rather than conclusive, given the cross-sectional, self-report design. No causal inferences can be drawn.

## 1 Introduction

With increasing concern about college students’ mental health, understanding how students regulate academic-related psychological states has become an important research focus. Psychological detachment, originally defined as the ability to mentally disengage from work-related thoughts during non-working time (Sonnentag and Fritz, 2007), has been widely conceptualized as a recovery-related experience that supports psychological well-being. In academic contexts, psychological detachment is conceptualized as a recovery-related construct reflecting individuals’ ability to mentally disengage from academic demands, rather than a direct indicator of academic engagement or academic performance.

When extending this construct to academic settings, it is important to distinguish psychological detachment from maladaptive forms of reduced academic involvement. Such maladaptive states are typically characterized by reduced motivation, emotional withdrawal, and diminished participation in learning activities, whereas psychological detachment refers to temporarily disengaging from academic-related thoughts during off-time to facilitate recovery. Lower levels of psychological detachment may reflect sustained cognitive engagement, insufficient recovery, or academic-related rumination. Accordingly, psychological detachment is treated as a recovery-related indicator whose functional meaning may be context-dependent rather than uniformly adaptive or maladaptive. Conflating psychological detachment with maladaptive withdrawal may contribute to conceptual ambiguity.

Previous research has shown that psychological detachment is positively associated with well-being and recovery outcomes (Biddle et al., 2019; Chen et al., 2025), while physical exercise has been linked to emotional regulation and stress-related processes (Herbert et al., 2020; Biddle et al., 2019). In the present study, exercise is conceptualized as exercise commitment, emphasizing motivational investment and persistence rather than objective activity levels. Despite this growing body of research, limited attention has been paid to how exercise-related dispositions relate to psychological detachment in academic contexts, particularly in non-Western settings. Importantly, as psychological detachment is operationalized here as a recovery-related construct, the observed associations should be interpreted as reflecting underlying processes rather than a single, clearly defined functional outcome.

From a mechanistic perspective, self-identity and sense of belonging may represent a theoretically grounded but not uniquely specified sequential pathway linking exercise commitment and psychological detachment. Self-identity reflects individuals’ self-perceptions and role integration (Drummond, 2021), which may shape subsequent social connectedness and belonging. Sense of belonging, in turn, has been associated with patterns of cognitive engagement and regulation in academic contexts (Korpershoek et al., 2020). However, given the cross-sectional design of the present study, this proposed sequence should be interpreted as one plausible configuration rather than a uniquely specified causal or temporal ordering among variables. Alternative sequences may also be consistent with the observed data.

Accordingly, this study addresses three gaps. First, at the construct level, it clarifies the conceptualization of psychological detachment within academic contexts, emphasizing its role as a recovery-related construct with context-dependent meaning. Second, at the analytical level, it examines a sequential mediation model involving self-identity and sense of belonging, with an emphasis on identifying statistical associations rather than establishing causal directionality. Third, at the cultural level, it explores these relationships within a collectivist context, where social connectedness may play a more central role in shaping psychological processes.

Based on these considerations, this study examines the association between exercise commitment and psychological detachment among Chinese college students and tests a chain mediation model involving self-identity and sense of belonging. The analysis focuses on statistically supported relationships derived from cross-sectional data, and the findings are interpreted as indicative of potential mechanisms rather than definitive evidence of causal processes or functional outcomes. Accordingly, the functional meaning of psychological detachment in academic contexts remains to be further clarified.

## 2 Theoretical foundations

This study develops an integrated theoretical framework in which Social Cognitive Theory, Self-Determination Theory, and Social Support Theory are jointly used to examine the associations among exercise commitment, self-identity, sense of belonging, and psychological detachment. The proposed framework reflects a theoretically informed but not uniquely specified ordering among these constructs, acknowledging that alternative configurations may also be consistent with the data. Rather than treating these theories as parallel perspectives, they are integrated into a coherent framework in which each contributes to understanding different aspects of the relationships examined, without implying a fixed or causally confirmed or temporally ordered sequence.

### 2.1 Integrated theoretical mapping

Drawing on Social Cognitive Theory, Self-Determination Theory, and Social Support Theory, this study develops a theoretically informed framework to examine the associations among exercise commitment, self-identity, sense of belonging, and psychological detachment. Social Cognitive Theory provides a basis for understanding how exercise commitment, as a sustained motivational orientation embedded in repeated behavioral contexts, is associated with the development of self-perceptions through ongoing interactions among behavior, cognition, and environmental feedback (Schunk and DiBenedetto, 2020). Self-Determination Theory further informs the association between self-identity and sense of belonging by emphasizing the role of basic psychological needs, particularly relatedness (Guay, 2022), suggesting that a more coherent sense of self may be linked to individuals’ orientation toward meaningful social connections. In parallel, Social Support Theory offers a perspective for interpreting how sense of belonging relates to psychological detachment, as social relationships provide emotional and interpersonal resources (Walton and Cohen, 2011) that may be associated with patterns of cognitive engagement during off-time. Taken together, these perspectives are integrated into a coherent but not uniquely specified framework, in which the ordering among constructs is theoretically informed yet open to alternative configurations, and should not be interpreted as a fixed or causally confirmed sequence.

### 2.2 Theoretical integration and model positioning

The three theoretical perspectives are integrated into a theoretically informed framework in which each theory is aligned with different aspects of the proposed relationships among exercise commitment, self-identity, sense of belonging, and psychological detachment. Specifically, Social Cognitive Theory provides a basis for understanding the association between behavioral engagement (exercise commitment) and self-perception (self-identity); Self-Determination Theory informs the association between self-perception and social connectedness (sense of belonging); and Social Support Theory offers a perspective on how social connectedness relates to cognitive regulation (psychological detachment). The proposed ordering among these constructs is theoretically informed but not uniquely specified, and alternative configurations (e.g., belonging preceding identity or direct associations between exercise commitment and psychological detachment) may also be consistent with the data. In this framework, identity-related processes are conceptualized as one plausible analytical pathway through which behavioral engagement may be associated with social and cognitive outcomes, without implying a fixed, causally confirmed, or temporally ordered sequence. Accordingly, the model is intended to reflect a structured yet non-exclusive representation of these associations, rather than a definitive or directionally established process.

## 3 Literature review and research hypotheses

### 3.1 Exercise commitment and psychological detachment

A growing body of research suggests that physical exercise is associated with various aspects of psychological functioning among college students, including emotional regulation and stress-related processes (Herbert, 2022; Biddle et al., 2019). In the present study, physical exercise is conceptualized as exercise commitment, which reflects individuals’ sustained motivational investment and persistence rather than the frequency or intensity of behavior. Compared with objective activity measures, this construct captures the psychological orientation underlying continued engagement in exercise contexts.

Psychological detachment refers to individuals’ ability to mentally disengage from academic-related thoughts during non-study time and is generally regarded as a recovery-related experience (Sonnentag and Fritz, 2007). However, its interpretation in academic settings may be context-dependent. While higher detachment typically facilitates recovery, lower levels of detachment may reflect sustained cognitive engagement with academic tasks, insufficient recovery, or a combination of both. Importantly, the functional meaning of psychological detachment in this context remains context-dependent and should not be interpreted as uniformly adaptive or maladaptive.

Emerging evidence suggests that exercise-related engagement may be linked to broader patterns of self-regulation and cognitive persistence. Individuals with higher exercise commitment may develop structured routines and goal-oriented tendencies that extend beyond exercise contexts. Such tendencies may be associated with differences in how individuals cognitively disengage from academic activities during non-study periods. However, the direction and functional implications of this association remain theoretically open, given the dual interpretation of psychological detachment. Accordingly, the present study examines the association between exercise commitment and psychological detachment without presuming a fixed functional interpretation.

*H1*: Exercise commitment is associated with psychological detachment among college students.

### 3.2 The mediating role of self-identity

Existing research suggests that physical exercise is associated with self-identity among college students, reflecting individuals’ perceptions of their own value, competence, and role (Drummond, 2021). In the present study, this construct is examined in relation to exercise commitment, which represents sustained motivational engagement and persistence in exercise contexts. Such engagement is associated with experiences including goal involvement, skill development, and social feedback, all of which may relate to variations in self-perception (Huang, 2024). Empirical evidence generally supports a positive association between exercise-related engagement and self-identity, although these relationships remain correlational in nature.

From a functional perspective, self-identity may be linked to patterns of cognitive and emotional regulation in academic contexts. Individuals with a more stable sense of self may differ in how they engage with or disengage from academic-related thoughts during non-study periods. However, the direction and functional implications of this relationship remain unclear, particularly given that lower psychological detachment may reflect sustained engagement, insufficient recovery, or a combination of both. Although limited research has directly examined self-identity in this context, it may be considered as a theoretically plausible analytical pathway within a multi-level perspective linking behavioral engagement and psychological processes. Accordingly, self-identity is treated as a potential mediating variable in the association.

*H2*: Self-identity is proposed as a mediator in the association between exercise commitment and psychological detachment among college students.

### 3.3 Mediating role of sense of belonging

Sense of belonging refers to individuals’ perceived experience of interpersonal connection and inclusion rather than a motivational need state (He, 2023). Within academic contexts, it is associated with perceived relatedness and social integration. Existing research suggests that a stronger sense of belonging is associated with various aspects of psychological functioning, including emotional stability and patterns of cognitive engagement (Allen et al., 2022). In the present study, exercise is conceptualized as exercise commitment, and higher levels of such commitment are associated with increased involvement in socially embedded activity contexts, which may provide opportunities for interaction and perceived relatedness (Patterson et al., 2021; Andersen et al., 2019).

Sense of belonging may also be associated with how individuals engage with or disengage from academic-related thoughts during non-study periods. Individuals with a stronger sense of belonging may differ in patterns of cognitive involvement or disengagement; however, the direction and functional implications of this relationship remain unclear. Given the context-dependent nature of psychological detachment, this association should not be interpreted as uniformly adaptive or maladaptive. Although limited research has directly examined sense of belonging in this context, it may be considered as a theoretically plausible analytical pathway within a broader framework linking social and psychological processes. Accordingly, sense of belonging is treated as a potential mediating variable in this association.

*H3*: Sense of belonging is proposed as a mediator in the association between exercise commitment and psychological detachment among college students.

### 3.4 The chain mediating roles of self-identity and sense of belonging

Self-identity and sense of belonging are closely related yet conceptually distinct constructs, and their directional relationship remains theoretically debated (Ragelienė, 2016). While Social Identity Theory suggests that group belonging may precede identity formation, Self-Determination Theory emphasizes that internal self-perception may provide a basis for relatedness. Given these differing perspectives, the relative ordering between self-identity and sense of belonging cannot be considered theoretically fixed.

In the present study, a sequence in which self-identity is positioned prior to sense of belonging is examined as one theoretically plausible configuration. From this perspective, self-identity may be understood as reflecting internal self-evaluative processes, whereas sense of belonging captures externally oriented social integration. This proposed ordering reflects a potential progression from “self-definition” to “social connection.” However, alternative sequences or reciprocal relationships remain equally plausible and cannot be ruled out.

**Figure 1:**
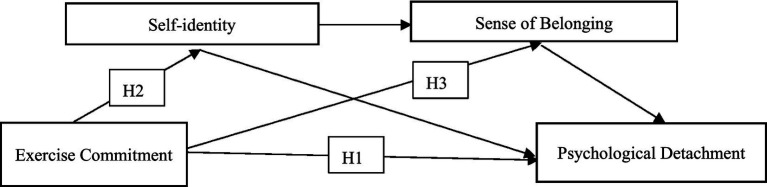
Research framework diagram.

Building on this framework, exercise commitment may be associated with identity-related experiences (e.g., competence, persistence), which may in turn relate to variations in sense of belonging within social and academic contexts. Sense of belonging may further be associated with patterns of cognitive engagement or disengagement during non-study periods. Importantly, given the context-dependent and functionally ambiguous nature of psychological detachment, the direction and implications of these associations remain open to multiple interpretations. Accordingly, this study examines a sequential analytical pathway involving self-identity and sense of belonging as a theoretically informed but empirically tentative representation, rather than a fixed or temporally ordered process (see Figure 1).

*H4*: Self-identity and sense of belonging are proposed as sequential mediators in the association between exercise commitment and psychological detachment, with the specified ordering representing one theoretically plausible configuration rather than a confirmed causal or temporal sequence.

## 4 Research subjects and methods

### 4.1 Research participants

A questionnaire survey was conducted among Chinese college students using the Exercise Commitment Questionnaire, Psychological Detachment Scale, Self-Identity Scale, and Sense of Belonging Scale. A total of 865 questionnaires were distributed. In contrast to the original description, the present study adopted a stratified multi-site sampling strategy combined with classroom-based convenience sampling, rather than strict probabilistic stratified random sampling. Mainland China was divided into five regions (East, South, Central, North, and West China) based on the National Bureau of Statistics classification. Within each region, 2–3 universities were selected based on accessibility and institutional cooperation (total = 13 institutions), rather than from a complete random sampling frame. Within each institution, participants were recruited during scheduled physical education (PE) classes, resulting in a cluster-based convenience sample of students currently enrolled in PE courses. This procedure enabled efficient data collection but may have excluded certain subgroups (e.g., senior students, postgraduate students, or those not enrolled in PE courses), thereby introducing potential sampling bias.

The distribution of valid samples across regions was as follows: East China 195 (22.54%), South China 175 (20.23%), Central China 165 (19.08%), North China 150 (17.34%), and West China 180 (20.81%). Participants were selected based on the following inclusion criteria: (1) currently enrolled university students; (2) engagement in physical exercise at least once per week. This criterion intentionally focuses on an “active student population” and excludes non-exercisers; therefore, the findings should be interpreted within this specific subgroup rather than generalized to all college students.

After data cleaning (removal of missing or invalid responses), 850 valid questionnaires were retained (response rate = 98.27%). Participants ranged in age from 17 to 25 years (M = 21.00, SD = 1.96), including 415 males (48.82%) and 435 females (51.18%). A *priori* power analysis using G*Power 3.1 (Cohen, 1992) indicated a minimum sample size of 88 (f^2^ = 0.15, *α* = 0.05, power = 0.90, four predictors). The final sample size (*N* = 850) substantially exceeded this requirement, ensuring adequate statistical power; however, statistical power does not eliminate potential sampling bias. This study was approved by the Institutional Review Board of Zhaoqing University (2025034). All participants provided informed consent prior to participation.

### 4.2 Research methods

#### 4.2.1 Psychometric methods

Exercise commitment scale

The present study employed the revised Physical Exercise Scale developed by Wu (2016), which is conceptually aligned with the construct of exercise commitment rather than actual exercise behavior. The scale was adapted from the Physical Exercise Commitment Intention Scale (Chen et al., 2006) and consists of 8 items across two dimensions: exercise commitment and exercise persistence, with four items in each dimension. Responses are recorded on a five-point Likert scale (1 = strongly disagree, 5 = strongly agree), with higher scores indicating a stronger motivational and attitudinal orientation toward exercise. Importantly, this instrument captures a psychological disposition toward exercise (i.e., commitment and persistence) rather than objective behavioral indicators such as frequency, intensity, or duration (e.g., as assessed by PARS-3 or IPAQ). Accordingly, in the present study, the independent variable is operationalized as exercise commitment, and all analyses and interpretations are based on this construct. Previous research has demonstrated acceptable reliability and validity of this scale in Chinese college student samples. In the current study, internal consistency was acceptable (Cronbach’s *α* = 0.742), and sampling adequacy was supported (KMO = 0.855). Confirmatory factor analysis indicated a good model fit (CFI = 0.991, TLI = 0.987, RMSEA = 0.023, SRMR = 0.025), supporting the construct validity of the scale in the present sample.

Self-identity scale

The present study employed the Self-Identity Scale originally developed by Ochse and Plug (1986) to assess the clarity, stability, and evaluative aspects of individuals’ self-concept. The scale consists of 19 items, with responses recorded on a 4-point Likert scale (1 = strongly disagree, 4 = strongly agree). Total scores are calculated by summing all item responses, with higher scores indicating a more developed and stable sense of self-identity. Previous research has demonstrated acceptable reliability and validity of this scale in Chinese university student samples (Jin, 2022). In the present study, internal consistency was acceptable (Cronbach’s *α* = 0.789), and sampling adequacy was supported (KMO = 0.902). Confirmatory factor analysis indicated a good model fit (CFI = 1.000, TLI = 1.005, RMSEA = 0.000, SRMR = 0.020), supporting the construct validity of the scale in the present sample.

Sense of belonging inventory

The present study employed the Sense of Belonging Inventory (SOBI; Hagerty and Patusky, 1995) to assess participants’ experienced sense of belonging as a psychological state of interpersonal connectedness. Accordingly, the mediator in this study is conceptualized as sense of belonging, reflecting a perceived and fulfilled state rather than a motivational construct. The SOBI consists of 18 items across two dimensions: psychological state belongingness and experiential belongingness. Responses are recorded on a 4-point Likert scale, with higher scores indicating a stronger sense of belonging. This instrument captures experienced belongingness as a psychological state, rather than motivational processes related to unmet sense of belonging. Therefore, all theoretical interpretations in the present study are aligned with a belonging-based perspective. Previous research has demonstrated acceptable reliability and validity of the SOBI in Chinese college student samples. In the current study, internal consistency was acceptable (Cronbach’s *α* = 0.714), and sampling adequacy was supported (KMO = 0.839). Confirmatory factor analysis indicated a good model fit (CFI = 0.977, TLI = 0.974, RMSEA = 0.014, SRMR = 0.024), supporting the construct validity of the scale in the present sample.

Psychological detachment scale

Psychological detachment was originally conceptualized in occupational settings as individuals’ ability to mentally distance themselves from work-related thoughts during non-working time (Sonnentag and Fritz, 2007). In the present study, this construct is adapted to the academic context and refers to students’ ability to mentally distance themselves from study-related thoughts during off-time, representing a form of recovery experience. The psychological detachment subscale of the Recovery Experience Questionnaire (REQ) was employed. This subscale consists of four items and has been widely used in prior research as a brief and validated measure of detachment. Responses are recorded on a 5-point Likert scale (1 = strongly disagree to 5 = strongly agree), with higher scores indicating greater psychological detachment and more effective recovery from academic demands. In the present sample, the scale demonstrated acceptable internal consistency (Cronbach’s *α* = 0.761) and sampling adequacy (KMO = 0.884). Confirmatory factor analysis indicated a good model fit (CFI = 1.000, TLI = 1.022, RMSEA = 0.000, SRMR = 0.004), supporting the construct validity of the scale in the present sample.

Importantly, psychological detachment in this study is conceptualized as a recovery-related construct rather than a maladaptive form of withdrawal. Accordingly, interpretations of the results are aligned with this conceptualization. Nevertheless, the use of a relatively brief subscale may limit the comprehensiveness of construct measurement. Future research is encouraged to employ more extensive and academically contextualized instruments to further validate the findings.

#### 4.2.2 Mathematical statistics methods

Statistical analyses were conducted using SPSS 26.0 and AMOS.

First, descriptive statistics and group difference tests were performed for demographic variables, exercise commitment, psychological detachment, self-identity, and sense of belonging. Second, to assess potential common method bias, both Harman’s single-factor test and confirmatory factor analysis (CFA) with an unmeasured latent method factor were employed. The CFA analyses were conducted using AMOS following established recommendations. Third, Pearson’s correlation analysis was performed to examine the associations among exercise commitment, self-identity, sense of belonging, and psychological detachment. Finally, Hayes’ PROCESS macro (Model 6) was applied to test the chain mediation model, using bias-corrected bootstrap confidence intervals based on 5,000 resamples. All indirect effects are reported as completely standardized estimates. Age and gender were included as control variables (covariates) in all regression models to to account for their potential association with the study variables.

## 5 Results

### 5.1 Descriptive statistical analysis of exercise commitment, psychological detachment, self-identity, and sense of belonging

It should be noted that exercise commitment, as examined in this study, reflects a motivational–attitudinal construct rather than actual physical activity behavior. Before examining gender differences, measurement invariance across gender was assessed using multi-group confirmatory factor analysis (CFA), including configural, metric, and scalar models. The baseline model demonstrated acceptable fit, indicating a comparable factor structure across groups. When factor loadings and item intercepts were constrained, changes in model fit remained minimal (ΔCFI < 0.01, ΔRMSEA < 0.015), supporting configural, metric, and scalar invariance across gender groups. On this basis, comparisons between male and female participants are considered meaningful and interpretable. The results presented in Tables 1, 2 indicate that exercise commitment and self-identity differ significantly by gender (*p* < 0.05), whereas psychological detachment and sense of belonging do not show statistically significant differences (*p* > 0.05).

**Table 1:** Gender differences in exercise commitment, psychological detachment, self-identity, and sense of belonging.

Variable	Gender	Number (%)	*M*	*SD*	*t*	*p*
Exercise commitment	Male	415 (48.82%)	27.535	4.841	3.624	0.000
	Female	435 (51.18%)	26.287	5.179		
	Total	850				
Psychological detachment	Male	415 (48.82%)	12.205	2.774	0.164	0.870
	Female	435 (51.18%)	12.172	2.972		
	Total	850				
Self-identity	Male	415 (48.82%)	54.422	7.809	−2.733	0.006
	Female	435 (51.18%)	55.878	7.726		
	Total	850				
Sense of belonging	Male	415 (48.82%)	44.911	6.690	−0.625	0.532
	Female	435 (51.18%)	45.202	6.904		
	Total	850				

**Table 2:** Manifestations of age differences in exercise commitment, psychological detachment, self-identity, and sense of belonging.

Variable	Age	Number (%)	M	SD	F	*p*
Exercise commitment	17	40 (4.71%)	25.425	4.898	2.194	0.026
	18	55 (6.47%)	26.873	5.433		
	19	96 (11.29%)	27.156	4.705		
	20	148 (17.41%)	27.176	4.968		
	21	163 (19.18%)	27.264	4.857		
	22	151 (17.76%)	27.695	5.068		
	23	116 (13.65%)	26.043	5.108		
	24	46 (5.41%)	25.196	5.472		
	25	35 (4.12%)	26.629	5.281		
	Total	850	26.896	5.052		
Psychological detachment	17	40 (4.71%)	11.6	2.619	3.221	0.001
	18	55 (6.47%)	12.345	3.534		
	19	96 (11.29%)	11.854	2.92		
	20	148 (17.41%)	11.912	2.725		
	21	163 (19.18%)	12.043	2.798		
	22	151 (17.76%)	11.874	2.76		
	23	116 (13.65%)	12.879	2.741		
	24	46 (5.41%)	13.609	2.687		
	25	35 (4.12%)	12.571	3.284		
	Total	850	12.188	2.875		
Self-identity	17	40 (4.71%)	55.375	6.217	0.842	0.565
	18	55 (6.47%)	55.164	8.548		
	19	96 (11.29%)	54.49	8.185		
	20	148 (17.41%)	55.838	7.663		
	21	163 (19.18%)	54.675	7.679		
	22	151 (17.76%)	55.603	7.803		
	23	116 (13.65%)	54.612	7.832		
	24	46 (5.41%)	54.326	8.13		
	25	35 (4.12%)	57.314	7.718		
	Total	850	55.167	7.796		
Sense of belonging	17	40 (4.71%)	43.5	6.891	1.564	0.132
	18	55 (6.47%)	44.091	6.802		
	19	96 (11.29%)	45.958	6.898		
	20	148 (17.41%)	45.588	6.691		
	21	163 (19.18%)	44.577	7.068		
	22	151 (17.76%)	45.232	6.304		
	23	116 (13.65%)	45.405	6.704		
	24	46 (5.41%)	43.13	7.29		
	25	35 (4.12%)	46.571	6.853		
	Total	850	45.06	6.798		

With respect to age, exercise commitment and psychological detachment showed statistically significant overall differences across age groups (*p* < 0.05). However, the effect sizes were relatively small (e.g., *F* = 2.194), indicating weak effects. *Post hoc* comparisons were not further interpreted, as appropriate family-wise error correction (e.g., Bonferroni or Tukey) was not applied. Therefore, these age-related differences should be interpreted with caution and are not emphasized in subsequent analyses. In terms of descriptive trends, male participants reported higher scores on exercise commitment and psychological detachment, whereas female participants showed higher levels of self-identity and sense of belonging. Given that measurement invariance was established, these observed differences are more likely to reflect group-level variations rather than measurement artifacts while their potential associations with model estimates are further examined in subsequent robustness analyses.

### 5.2 Common method Bias test

As all data in this study were collected via self-report questionnaires, common method bias (CMB) may be a potential concern. Harman’s single-factor test was first conducted as a preliminary diagnostic. The results showed that 12 factors had eigenvalues greater than 1, and the first factor accounted for 16.52% of the total variance, which is below the commonly used threshold of 40%, suggesting that no single factor dominated the variance structure.

Given the limitations of Harman’s test, a more rigorous approach was subsequently applied following Podsakoff et al. (2003). Specifically, a confirmatory factor analysis (CFA) with an unmeasured latent method factor was conducted using AMOS. A common latent factor was added to the measurement model, with all observed items loading on both their respective constructs and the latent method factor. The baseline measurement model demonstrated acceptable fit (χ^2^/df = 1.630, CFI = 0.985, TLI = 0.982, RMSEA = 0.027). After including the latent method factor, the model fit showed only marginal improvement (χ^2^/df = 1.580, CFI = 0.987, TLI = 0.984, RMSEA = 0.026), with changes in fit indices below recommended thresholds (ΔCFI < 0.01, ΔRMSEA < 0.015). In addition, factor loadings on the method factor were relatively low. These results suggest that common method variance is unlikely to substantially bias the observed relationships, although it cannot be entirely ruled out.

### 5.3 Correlation analysis of exercise commitment, psychological detachment, self-identity, and sense of belonging

Table 3 shows that exercise commitment is negatively correlated with psychological detachment and positively correlated with self-identity and sense of belonging. Psychological detachment is negatively associated with both self-identity and sense of belonging, while self-identity is positively related to sense of belonging. Overall, the variables are significantly correlated with each other, indicating that the basic conditions for further mediation analysis are met.

**Table 3:** Correlation analysis statistics for exercise commitment, psychological detachment, self-identity, and sense of belonging.

Variable	M ± SD	Exercise commitment	Psychological detachment	Self-identity	Sense of belonging
Exercise Commitment	26.896 ± 5.052	1			
Psychological Detachment	12.188 ± 2.875	−0.501**	1		
Self-identity	55.167 ± 7.796	0.583**	−0.570**	1	
Sense of Belonging	45.060 ± 6.798	0.558**	−0.542**	0.633**	1

*N* = 850. **p < 0.01.

### 5.4 Testing the mediation model

A chain mediation model was estimated using PROCESS Model 6 (5,000 bootstrap samples), with exercise commitment as the independent variable, self-identity and sense of belonging as mediators, and psychological detachment as the outcome variable. All reported coefficients are unstandardized regression coefficients (B).

**Figure 2:**
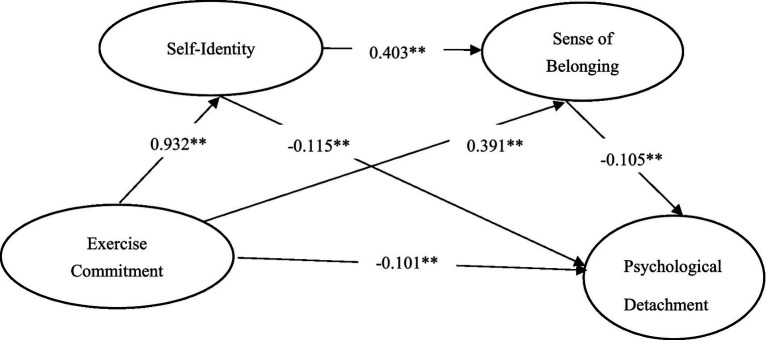
Chain mediation model of self-identity and sense of belonging in the relationship between exercise commitment and psychological detachment.

The results (Table 4; Figure 2) indicate that exercise commitment is negatively associated with psychological detachment (*B* = −0.101, *p* < 0.001), and positively associated with self-identity (*B* = 0.932, *p* < 0.001) and sense of belonging (*B* = 0.391, *p* < 0.001). Self-identity is positively associated with sense of belonging (*B* = 0.403, *p* < 0.001), whereas both self-identity (*B* = −0.115, *p* < 0.001) and sense of belonging (*B* = −0.105, *p* < 0.001) are negatively associated with psychological detachment. The total effect is *B* = −0.289 (*p* < 0.001). The model explains 36.8% of the variance in self-identity, 45.6% in sense of belonging, and 41.4% in psychological detachment, indicating a moderate to relatively high level of explained variance.

**Table 4:** Regression results for the serial mediation model.

Variables	Self-identity		Sense of belonging		Psychological detachment		Total effect	
	B	t	B	t	B	t	B	t
Exercise commitment	0.932	21.935**	0.391	9.069**	−0.101	−5.089**	−0.289	−17.074**
Self-identity			0.403	14.487**	−0.115	−8.387**		
Sense of belonging					−0.105	−6.975**		
*R* ^2^	0.368		0.456		0.414		0.265620	
F	164.345		176.936		119.256		101.997	

B, unstandardized regression coefficient. **p < 0.001.

Given the relatively high *R*^2^ for psychological detachment, additional tests of discriminant validity were conducted. The Fornell–Larcker criterion indicated that the square roots of the average variance extracted (AVE) for all constructs exceeded their inter-construct correlations. In addition, the heterotrait–monotrait ratio (HTMT) values were all below the recommended threshold (e.g., 0.85), with values ranging from 0.63 to 0.76, further supporting discriminant validity. Taken together, these results suggest that the observed relationships are unlikely to be substantially accounted for by construct overlap or common measurement issues.

### 5.5 Indirect effects and alternative model analysis

Indirect effects were examined using bias-corrected Bootstrap confidence intervals based on 5,000 resamples. As shown in Table 5, the total indirect effect was statistically significant (*B* = −0.188, Boot SE = 0.014, 95% CI [−0.218, −0.160]), as the confidence interval did not include zero. Three indirect pathways were observed. First, the pathway through self-identity (exercise commitment → self-identity → psychological detachment) was significant (*B* = −0.107, 95% CI [−0.134, −0.081]), accounting for 37.02% of the total effect. Second, the pathway through sense of belonging (exercise commitment → sense of belonging → psychological detachment) was also significant (*B* = −0.041, 95% CI [−0.057, −0.028]), accounting for 14.19% of the total effect. Third, the sequential indirect pathway through both self-identity and sense of belonging (exercise commitment → self-identity → sense of belonging → psychological detachment) was significant (*B* = −0.040, 95% CI [−0.053, −0.028]), accounting for 13.84% of the total effect. Both unstandardized coefficients (B) and completely standardized indirect effects (*β*) are reported in Table 5 to facilitate interpretation.

**Table 5:** Indirect effects of the serial mediation model.

Type of effect	Effect (B)	Standardized effect (*β*)	Boot SE	95% Bootstrap CI		Proportion (%)
				LL	UL	
Total effect	−0.289	—	0.017	−0.322	−0.255	100%
Direct effect	−0.101	—	0.02	−0.14	−0.062	34.95%
Exercise Commitment - Self-Identity - Psychological Detachment	−0.107	−0.187	0.014	−0.134	−0.081	37.02%
Exercise Commitment - Sense of Belonging - Psychological Detachment	−0.041	−0.072	0.007	−0.057	−0.028	14.19%
Exercise Commitment - Self-Identity -Sense of Belonging - Psychological Detachment	−0.041	−0.070	0.006	−0.053	−0.028	13.84%
Total indirect effect	−0.188	−0.329	0.014	−0.218	−0.16	65.05%

Notably, the total indirect effect accounted for 65.03% of the total effect, exceeding the direct effect (34.97%), indicating that the association between exercise commitment and psychological detachment is primarily transmitted through the mediating mechanisms. To examine alternative causal orderings, a reversed serial mediation model (exercise commitment → sense of belonging → self-identity → psychological detachment) was additionally tested. The results indicated that the reversed model also yielded a significant total indirect effect (effect = −0.188, 95% CI [−0.217, −0.160]) with comparable explanatory power (*R*^2^ = 0.414). The completely standardized total indirect effect was −0.329 (95% CI [−0.376, −0.286]).

However, despite similar statistical performance, the hypothesized model provides a more theoretically coherent representation of the relationships among variables, particularly in capturing the sequential progression from identity-related processes to social relational experiences. In contrast, the reversed ordering implies a less conceptually grounded pathway. Taken together, these findings are consistent with the proposed sequential mediation structure, while acknowledging that alternative model specifications cannot be entirely ruled out.

### 5.6 Robustness check with control variables

To assess the robustness of the findings, age and gender were included as control variables in the PROCESS Model 6 analysis. After controlling for these variables, the direction, magnitude, and statistical significance of both the direct and indirect effects remained largely unchanged. The serial mediation pathways through self-identity and sense of belonging continued to be significant, and the relative contribution of the indirect effects showed little variation compared to the main model. These results indicate that the observed associations are relatively stable after accounting for basic demographic characteristics. However, this robustness should be interpreted cautiously, as the cross-sectional design and the potential nesting of participants within institutions and classes may affect the precision of standard errors and parameter estimates. Therefore, the present findings should be regarded as providing preliminary rather than definitive evidence for model stability.

## 6 Discussion

### 6.1 Exercise commitment and psychological detachment

The present study found that exercise commitment was negatively associated with psychological detachment among college students. This finding requires careful interpretation, as psychological detachment is conceptualized as a recovery-related process involving mental disengagement from academic demands during off-time (Sonnentag and Fritz, 2007), rather than a direct indicator of academic engagement or performance. Accordingly, lower levels of detachment cannot be interpreted as uniformly adaptive or maladaptive.

A key issue highlighted by these findings is the context-dependent nature of psychological detachment in academic settings. Reduced detachment may reflect sustained cognitive engagement or insufficient recovery, and the present study does not provide sufficient evidence to distinguish between these possibilities. Therefore, psychological detachment is treated here as a recovery-related indicator with potentially mixed functional implications rather than a unidirectional outcome.

From this perspective, individuals with higher exercise commitment may exhibit stronger self-regulatory tendencies and more consistent goal-directed orientations that extend beyond exercise contexts. These tendencies may be associated with a reduced likelihood of fully disengaging from academic-related thoughts during non-study periods. However, this interpretation should be considered as one plausible explanation rather than a confirmed mechanism, particularly because the present study does not directly assess academic engagement, cognitive persistence, or rumination. As such, the observed association cannot be conclusively attributed to enhanced academic involvement.

Alternative explanations should also be considered. Given the cross-sectional design, the observed association cannot be interpreted as directional. It is equally plausible that individuals who remain cognitively engaged with academic tasks are more likely to maintain structured activities such as exercise, or that both variables are influenced by unmeasured third factors (e.g., personality traits, academic pressure, or self-discipline). In addition, the relatively strong association may partly reflect shared method variance among self-reported constructs. Although similar patterns have been reported in prior research (Trigueros et al., 2019; Koch et al., 2024), the strength and interpretation of this relationship should be treated with caution.

Taken together, these findings suggest that the association between exercise commitment and psychological detachment is best understood as reflecting multiple possible processes rather than a single explanatory pathway. In this sense, the present results should be interpreted as indicative of potential underlying processes rather than as evidence of a specific causal or functional mechanism.

In addition, gender differences were observed in exercise commitment and self-identity; however, these findings were not hypothesized *a priori* and should be interpreted as exploratory. While gender role theory may offer one possible explanation, such interpretations remain tentative and require further empirical validation. The absence of gender differences in psychological detachment and sense of belonging further suggests that these constructs may be more strongly shaped by contextual or situational factors than by gender alone. Future research should examine these patterns using pre-registered hypotheses and more rigorous research designs.

### 6.2 Mediating role of self-identity

The findings indicate that self-identity is systematically associated with the relationship between exercise commitment and psychological detachment. Rather than supporting a definitive causal mechanism or a uniquely determined mediation sequence, this pattern is more appropriately interpreted as a structured configuration of associations linking behavioral engagement, self-related cognition, and cognitive states. Exercise commitment represents a sustained form of motivational investment embedded in repeated experiences of goal pursuit, perceived competence, and social participation, which may be associated with the development of a more coherent and stable sense of self.

From a theoretical perspective, self-identity can be conceptualized as an internalized cognitive structure through which individuals organize their experiences and regulate goal-directed behavior. Compared to momentary cognitive states such as psychological detachment, self-identity operates at a more stable, intrapersonal level, which makes it theoretically plausible—but not empirically established in the present study—that it may occupy a relatively prior position in linking sustained behavioral engagement with variations in cognitive involvement in academic contexts. Importantly, this assumption is not uniquely supported by the data. In this sense, self-identity may function as an organizing framework associated with patterns of continued cognitive activation. However, this association should be interpreted cautiously, as psychological detachment is treated as a recovery-related indicator with context-dependent meaning rather than a direct measure of academic functioning.

Crucially, the mediating role identified in this study does not imply a single or confirmed psychological mechanism. Instead, the observed pattern may reflect multiple competing or overlapping processes, including self-regulation, goal persistence, or broader dispositional tendencies, which are not directly measured in the present study. In addition, because psychological detachment is assessed as a recovery-related construct rather than a direct indicator of academic engagement, cognitive persistence, or learning involvement, the association between self-identity and detachment cannot be interpreted as evidence of enhanced academic functioning.

At the same time, caution is warranted in interpreting this mediating role. The cross-sectional design precludes conclusions regarding temporal ordering, and alternative explanations remain plausible. For instance, individuals who maintain persistent cognitive engagement with academic tasks may gradually develop a more salient or stable sense of self, suggesting a potential reverse pathway. Moreover, the alternative serial mediation model tested in this study yielded comparable indirect effects and explanatory power, indicating that the proposed ordering of variables should be understood as one plausible configuration rather than an empirically confirmed sequence. In addition, the use of self-report measures for all variables raises the possibility of shared method variance, particularly given the conceptual proximity between self-identity and cognitive engagement-related constructs.

Taken together, these findings should be interpreted as indicating a theoretically meaningful but non-exclusive and non-directional pathway, rather than as evidence of a fixed, causal, or temporally ordered process. Future research using longitudinal or experimental designs is needed to clarify temporal precedence and to more rigorously test competing model specifications.

### 6.3 Mediating role of sense of belonging

The findings indicate that sense of belonging is systematically associated with the relationship between exercise commitment and psychological detachment. Rather than reflecting a direct causal pathway, this pattern is better understood as a structured configuration in which socially embedded experiences co-vary with sustained behavioral engagement and patterns of cognitive involvement. Exercise commitment is typically situated within interactive contexts characterized by shared goals, cooperation, and group participation, which may be associated with individuals’ perceptions of social connection and acceptance.

From a theoretical perspective, sense of belonging reflects an interpersonal and relational evaluation of one’s position within a social environment. In contrast to self-identity, which represents an internalized and relatively stable self-concept, sense of belonging is more context-dependent and socially contingent. Although it is theoretically reasonable to consider that intrapersonal and interpersonal processes may be linked, the present findings do not provide empirical support for a fixed hierarchical ordering or directional sequence between self-identity and sense of belonging. Accordingly, the positioning of sense of belonging within the proposed chain should be understood as one possible conceptualization rather than an empirically established structure. In this sense, sense of belonging may be associated with how individuals experience and interpret their social environments, which in turn may relate to patterns of cognitive involvement in academic contexts. This association should be interpreted cautiously, as psychological detachment is treated as a recovery-related construct with context-dependent meaning rather than a direct indicator of academic functioning.

Importantly, the functional meaning of this association remains context-dependent. Psychological detachment in this study is interpreted as a recovery-related indicator, and its association with sense of belonging may reflect multiple possible processes rather than a single functional outcome. Moreover, because psychological detachment is operationalized as a recovery-related construct rather than a direct measure of academic engagement, cognitive persistence, or performance, the observed association cannot be taken as evidence of enhanced academic functioning.

At the same time, this mediating role should be interpreted with caution. The cross-sectional design precludes conclusions regarding temporal ordering, and alternative model specifications remain plausible. For instance, individuals with stronger pre-existing social connectedness may be more likely to engage in exercise and to maintain higher levels of cognitive involvement in academic contexts, suggesting potential reverse or parallel pathways. Furthermore, the alternative serial mediation model tested in this study yielded comparable indirect effects, indicating that the proposed ordering of variables is not uniquely supported by the data. In addition, the reliance on self-report measures and the conceptual overlap among social and psychological constructs may introduce shared variance, potentially inflating the observed associations.

The observed negative association between sense of belonging and psychological detachment should therefore be interpreted cautiously. One possible explanation is that stronger social connectedness is associated with greater involvement in socially and academically embedded activities, which may be linked to reduced disengagement during non-study periods. However, alternative interpretations are equally plausible, and this pattern should not be taken as evidence of a single underlying mechanism.

Taken together, these findings should be interpreted as reflecting a theoretically informed but non-exclusive configuration of associations, rather than evidence of a fixed, causal, or temporally ordered process. The interpretation presented here represents one of several plausible frameworks that require further empirical validation. Future research employing longitudinal or experimental designs is needed to clarify temporal precedence, compare competing model structures, and examine how contextual factors (e.g., type of activity or group environment) may shape these processes. Together with the role of self-identity, the present results point to a multi-level but not uniquely specified framework linking behavioral engagement to cognitive states through both intrapersonal and interpersonal processes.

### 6.4 Chain mediation effect of self-identity and sense of belonging

The findings point to a sequentially organized pattern in which self-identity and sense of belonging are jointly associated with the relationship between exercise commitment and psychological detachment. However, this pattern should not be interpreted as evidence of a confirmed or uniquely specified sequence. Rather, it is more appropriately understood as a theoretically grounded configuration that integrates behavioral engagement, intrapersonal cognition, and interpersonal experience within a single framework. In this sense, the proposed sequence serves as a conceptual model for organizing the observed associations, rather than as an empirically established representation of underlying psychological processes.

From a theoretical standpoint, the ordering of self-identity and sense of belonging reflects a distinction between internal self-organization and externally oriented relational evaluation. Self-identity represents an internalized, relatively stable cognitive structure through which individuals interpret experiences and regulate goal-directed behavior, whereas sense of belonging reflects a context-dependent perception of one’s social embeddedness and relational positioning. While this distinction provides a theoretically coherent rationale for the proposed ordering, it does not constitute empirical evidence that this sequence operates in the manner specified in the present model. Importantly, the present data do not support a uniquely specified or directionally confirmed ordering between these variables.

Accordingly, the proposed chain can be understood as a conceptual sequence linking behavioral engagement (exercise commitment), intrapersonal organization (self-identity), interpersonal integration (sense of belonging), and variations in cognitive states related to academic activity, as reflected in psychological detachment. Importantly, this sequence should be interpreted as one possible analytical representation rather than a definitive or exclusive pathway. Consistent with previous sections, psychological detachment is treated as a recovery-related construct with context-dependent meaning, and its association with other variables should not be interpreted as reflecting a single functional outcome.

At the same time, this sequential configuration should be interpreted with considerable caution. The cross-sectional design precludes conclusions regarding temporal precedence, and multiple alternative model specifications remain plausible. Notably, the alternative serial mediation model examined in this study yielded comparable indirect effects and explanatory power, indicating that the proposed ordering is not uniquely supported by the data and that reverse or parallel pathways may be equally plausible. In addition, the reliance on self-report measures and the conceptual relatedness among the constructs may introduce shared variance, potentially inflating the observed indirect effects. Furthermore, because psychological detachment is assessed as a recovery-related construct rather than a direct measure of academic engagement or cognitive functioning, the interpretation of the overall chain in terms of academic processes remains limited. These findings provide support for a sequential analytical pathway involving self-identity and sense of belonging within the proposed framework, while emphasizing that the ordering is theoretically informed rather than empirically established.

Taken together, the present findings are better understood as reflecting a theoretically informed but non-exclusive configuration of associations, rather than evidence of a fixed, causal, or temporally ordered mechanism. The interpretation offered here represents one of several plausible frameworks that require further empirical validation. Future research employing longitudinal, experimental, or cross-lagged designs is needed to adjudicate among competing models and clarify the conditions under which different configurations may be supported.

Overall, the present findings are best understood as reflecting a theoretically grounded but non-exclusive framework of associations, rather than evidence of a fixed, causal, or temporally ordered process.

## 7 Practical significance

The present findings provide a theoretically informed basis for considering how different levels of psychological processes may be relevant in exercise-related contexts. The relatively larger indirect association observed through self-identity (37.02%), compared to the sequential indirect pathway involving sense of belonging (13.84%), suggests that intrapersonal processes may represent a more prominent component within the overall pattern of associations, whereas interpersonal processes may play a complementary role. Importantly, these differences should be interpreted as indicative of relative salience within the observed model, rather than as prescriptive evidence for intervention prioritization.

Within this framework, exercise-based contexts may benefit from incorporating elements that are conceptually aligned with identity-related processes, such as goal-setting, self-monitoring, and competence-related feedback. These components are widely discussed in the literature as conditions associated with the development of more coherent self-perceptions and may support sustained psychological engagement in activity contexts. At the same time, these implications are theoretically derived and should be interpreted with appropriate caution.

Similarly, socially interactive formats (e.g., group-based or peer-supported activities) may provide contexts conducive to the development of a sense of belonging. Such formats may be particularly relevant in environments emphasizing social integration and shared participation, highlighting the potential role of interpersonal processes in shaping psychological experiences. However, the extent to which these elements produce specific outcomes remains to be further examined.

In addition, the temporal structure of participation may be relevant. More continuous and sustained engagement, rather than short-term or irregular involvement, may be more closely aligned with identity-related processes over time. It should be noted that exercise commitment in this study reflects a motivational–attitudinal orientation rather than objective behavioral participation, and therefore these implications should be interpreted within that scope.

Overall, the present findings highlight the potential value of considering both intrapersonal (e.g., self-identity) and interpersonal (e.g., sense of belonging) processes when designing exercise-related contexts for university students, particularly in relation to recovery-related psychological experiences. At the same time, given the cross-sectional and self-report nature of the data, these implications should be understood as theoretically informed considerations rather than definitive guidelines. Future research using longitudinal and experimental designs is needed to further evaluate their applicability across contexts. In addition, because participants were recruited from physical education classes and required to engage in regular exercise, the findings may be more applicable to physically active students rather than the general university population.

## 8 Research limitations and future directions

This study has several limitations that should be considered when interpreting the findings.

First, a central limitation concerns the conceptual positioning of psychological detachment. Although it was defined and measured as a recovery-related construct, its application in an academic context introduces potential ambiguity in interpretation. Psychological detachment is treated in this study as a recovery-related indicator rather than a direct measure of academic engagement or performance, and its functional meaning in academic settings remains context-dependent.

Second, limitations related to construct measurement should be acknowledged. Psychological detachment was assessed using a brief subscale, which may constrain the breadth of construct representation. In addition, the conceptual proximity among exercise commitment, self-identity, and sense of belonging raises the possibility of construct overlap. Combined with the use of a single measurement method, this may contribute to shared variance. Future research should incorporate multi-method approaches and more comprehensive measurement strategies to better distinguish related constructs.

Furthermore, the psychological detachment scale used in this study was adapted from a work-context measure to an academic context. Although prior research supports its applicability, additional validation, including confirmatory factor analysis (CFA) and detailed reporting of model fit indices, would further strengthen measurement transparency.

Third, the cross-sectional design imposes important limitations on the interpretation of structural relationships. Although a theoretically informed sequential model was proposed, the present findings do not provide empirical support for a uniquely determined or directionally confirmed mediation sequence. Multiple alternative models—including reversed, reciprocal, or parallel structures—remain plausible. Accordingly, the proposed chain should be interpreted as a theoretically informed but empirically tentative configuration.

Fourth, the sampling strategy may limit generalizability. By including only participants who engaged in exercise at least once per week and recruiting from physical education classes, the study may exclude less active individuals and reduce variability in exercise-related dispositions. The findings may therefore be more applicable to physically active university students rather than the general student population.

Fifth, all variables were assessed using self-report measures, which may introduce common method variance and perceptual bias. In addition, participants were nested within institutions and physical education classes, while the analyses were conducted at the individual level using PROCESS regression, which assumes independence of observations. Therefore, potential clustering effects cannot be fully ruled out and may influence standard errors and parameter estimates.

Taken together, these limitations indicate that the present study provides theoretically informed but preliminary insights rather than definitive evidence. In particular, the findings should not be interpreted as evidence of causal mechanisms or as a basis for directly guiding intervention practices without further empirical validation. Future research employing longitudinal, experimental, and multi-level designs is needed to clarify causal relationships, test competing models, and extend the generalizability of the findings.

## 9 Conclusion

This study examined the associations between exercise commitment and psychological detachment through the mediating roles of self-identity and sense of belonging within an academic context. The findings indicate that exercise commitment is associated with psychological detachment through both direct and indirect pathways, with self-identity representing a relatively more prominent pathway and sense of belonging playing a complementary role. However, these relationships should not be interpreted as evidence of a confirmed causal structure or a uniquely supported mediation sequence.

The study contributes to the literature by situating psychological detachment within a multi-level framework linking behavioral engagement, intrapersonal processes, and interpersonal experiences. In this study, psychological detachment is interpreted as a recovery-related construct with context-dependent meaning rather than a direct indicator of academic engagement or performance. Accordingly, its functional role in academic contexts remains unresolved.

These findings should be interpreted with appropriate caution. The cross-sectional design precludes conclusions regarding temporal ordering or causal direction, and alternative model specifications—including reversed or parallel pathways—remain plausible. Accordingly, the proposed relationships are best understood as theoretically informed but empirically tentative. Furthermore, because the study relies on self-report measures and assesses exercise commitment as a motivational–attitudinal construct rather than actual behavior, the findings do not provide direct evidence of behavioral outcomes or intervention effectiveness.

Taken together, this study provides theoretically grounded but preliminary insights into the relationships among exercise commitment, self-identity, sense of belonging, and psychological detachment in academic contexts. Future research using longitudinal, experimental, and multi-level approaches is needed to clarify causal mechanisms, compare competing models, and further specify the role of psychological detachment across contexts.

## Data Availability

The raw data supporting the conclusions of this article will be made available by the authors, without undue reservation.
