# Supplementary material for: The relationship between exercise commitment and psychological detachment from academic activities among Chinese college students: the chain mediating roles of self-identity and sense of belonging
Source: Front Psychol. 2026 Jul 30;17:1810232. doi: 10.3389/fpsyg.2026.1810232 (PMC13467968; doi:10.3389/fpsyg.2026.1810232)
Supplement: Supplementary file 1 [file Supplementary_file_1.DOCX]

**Volunteer informed consent**

**1.Project Name:**

The Relationship Between Physical Exercise and Psychological Detachment Among Chinese College Students: The Chain-Mediating Role of Self-Identity and Social Needs

**2.Research Purpose:**

This study is a joint research project conducted by several teachers from Zhaoqing University and cooperative universities.

Objective: To explore the relationship between physical exercise and psychological detachment among Chinese college students, and to examine whether self-identity and social needs play a significant chain-mediating role between them.

Methods and Participants: A stratified random sampling method will be adopted to survey Chinese college students aged between 17–25 years. The investigation will use the Physical Exercise Scale, Psychological Detachment Scale, Self-Identity Scale, and Social Needs Scale. Data will be analyzed using SPSS 21.0 software and the PROCESS macro program for mediation analysis prepared by Hayes.

**3.Research Framework:**

(1) To examine the predictive role of physical exercise on psychological detachment in college students;

(2) To test the mediating role of self-identity between physical exercise and psychological detachment;

(3) To test the mediating role of social needs between physical exercise and psychological detachment;

(4) To verify the chain-mediating role of self-identity and social needs in the relationship between physical exercise and psychological detachment.

We sincerely invite you to participate in this study and thank you for your valuable support in advancing sports and mental health research in Chinese universities.

**Impact on You:**

Your participation only involves filling out questionnaires. All collected data will be used solely for scientific research purposes. This study will not have any negative impact on you or your academic activities, and no personal data will be made public or used for non-research purposes.

**4.Concept Explanation:**

4.1 Physical Exercise

Physical exercise refers to regular and purposeful physical activities (such as swimming, running, ball games, etc.) carried out to enhance physical fitness, emotional wellbeing, and social interaction. Sufficient and moderate exercise can improve cognitive functioning, reduce stress, and promote positive psychological states.

4.2 Psychological Detachment

Psychological detachment refers to a mental state in which individuals emotionally withdraw from study or daily engagement, showing reduced interest, low motivation, and feelings of disconnection or belonging loss. Appropriate psychological recovery through exercise can alleviate psychological fatigue and improve emotional readiness and learning adaptation.

4.3 Self-Identity

Self-identity is an individual’s recognition and affirmation of self-value, social roles, and belonging. Positive sports experiences and achievement feedback contribute to enhancing one’s confidence and sense of purpose, helping college students better integrate their personal and social identities.

4.4 Social Needs

Social needs refer to the internal motivation of individuals to seek communication, support, and social belonging through interpersonal interactions. Participating in physical exercise often provides social opportunities, helps individuals establish relationships, develop teamwork, and meet emotional connection needs, thereby reducing feelings of isolation and detachment.

**Commitment:**

All research data will be strictly used for scientific academic research and publication purposes only. Personal identity information will be anonymized during data processing to ensure confidentiality. When filling in questionnaires, your real name will be replaced by an English letter code, and no personal information will be disclosed.

If you have any questions regarding the above terms, you may consult our research staff directly.

By signing below, you confirm that you have read and understood all of the above information and voluntarily agree to participate in this study.

Signature of the Volunteer (or Legal Guardian): ___________________________

This Project Research Group

Date: ________ Year ________ Month ________ Day
